# Evaluation of the Shale Oil Reservoir and the Oil Enrichment Model for the First Member of the Lucaogou Formation, Western Jimusaer Depression, Junggar Basin, NW China

**DOI:** 10.1021/acsomega.1c00756

**Published:** 2021-04-28

**Authors:** Boyang Wang, Bo Liu, Guoxiang Sun, Longhui Bai, Yaao Chi, Qi Liu, Miao Liu

**Affiliations:** † Key Laboratory of Continental Shale Hydrocarbon Accumulation and Efficient Development, Ministry of Education, Northeast Petroleum University, Daqing 163318, China; ‡ PetroChina Jilin Oilfield Company, Songyuan 138000, China; § CNPC Engineering Technology R&D Company Limited, Beijing 102206, China; ∥ Oil Production Engineering Research Institute, Daqing Oil Field Company, Daqing 163453, China

## Abstract

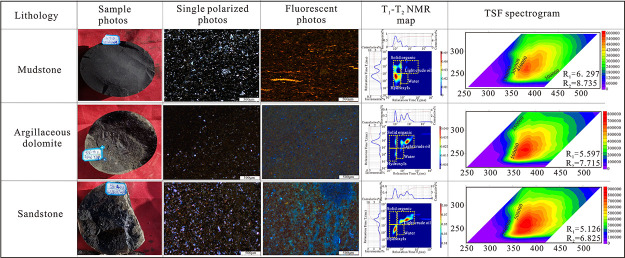

The Lucaogou Formation (Fm) in the Jimusaer depression is the first large-scale development of a terrigenous clastic sedimentary shale oil reservoir in China. Nearly one billion tons of shale oil resources have been discovered. However, the current exploration and development is concentrated in the eastern part of the sag. The limited geological understanding in the western area has restricted the prediction and development of “sweet spots” for shale oil. To help rectify this, we have studied the petrology, geochemistry, oil content, and pore properties of the second part of the first member (Mbr) of the Lucaogou Fm (P_2_l_1_^2^) in a typical well (Ji-X) in the western part of the sag. The results show that P_2_l_1_^2^ in the Jimusaer sag is a mixed fine-grained sedimentary system composed of sandstone, mudstone, and carbonate, which can be divided into seven types: dolomitic mudstone, calcareous mudstone, mudstone, mixed fine-grained rock, argillaceous limestone, sandstone, and argillaceous dolomite. The organic matter type of P_2_l_1_^2^ in well Ji-X is dominated by types I and II, and this is the best source rock in the whole exploration area of the Jimusaer sag. The overall oil saturation is relatively high, but the maturity of crude oil is low, and the overall oil quality is heavy, which is mainly controlled by the sedimentary environment and maturity of source rocks. Lithology and reservoir physical properties are the key to control oil content. The high-quality light oil reservoir lithology is argillaceous dolomite and sandstone. The higher the content of macropores and mesopores, the weaker the heterogeneity of the pore structure, and the better the oil content in the reservoir. There are four light oil sweet spots in the upper part (burial depth less than 4366 m), and there are excellent source rocks with high HI and high organic matter content near each sweet spot. This discovery of shale oil enrichment regularity will effectively guide the development of shale oil in continental lacustrine basins in other parts of the world.

## 1 Introduction

The discovery of Bakken shale oil in the United States has made the exploration and development of shale oil a hot topic.^1^ The geological resources of lacustrine shale oil are huge in China.^1^ To promote its industrial and commercial development is one of the important means to ensure national energy security. At present, there have been many achievements in shale oil-related research, but there are still many unsolved key problems related to shale oil exploitation. For example, the conventional classification schemes for shale pore types mostly adopt the decimal classification method suitable for coal proposed by Hodot in 1996 or the pore classification scheme for chemical synthetic materials proposed by IUPAC in 1972.^2^ However, the shale reservoir is obviously different from coal in the pore structure and mineral composition, and even more different from the chemical synthetic materials.^3^ Therefore, a pore classification scheme suitable for the shale oil reservoir is urgently needed.

A series of major breakthroughs have been made in shale oil exploration and development of the Lucaogou Formation (Fm) in the Jimusaer depression of the Junggar basin, and several wells have achieved commercial oil flow. The latest exploration shows that its reserve scale is one billion tons, and the exploitation potential is thus huge. However, the complex sedimentary environment and low thermal maturity have limited its economic development.^4,5^ Previous studies have focused on lithology and lithofacies,^6^ geochemical characteristics of source rocks,^7,8^ reservoir physical properties,^9,10^ shale oil properties,^11^ and oil-gas migration characteristics of Lucaogou Fm,^12^ but the research has been mostly concentrated in the eastern slope area, with little work in the western sag area.

During the deposition of Lucaogou Fm, the differences between the western sag and the eastern slope are as follows: (1) lake delta facies are developed in the eastern slope area, while semi-deep lake facies are developed in the western sag.^13^ Hence, rock types and properties are different. (2) The burial depth of the eastern slope area is shallower than in the western sag. The difference in the burial depth may lead to different temperature and pressure conditions and affected parameters, such as source rock maturity and reservoir physical properties. Therefore, the lithology, reservoir physical properties, source rocks, and oil content in the western sag are of great significance for expanding the exploration and development potential of lacustrine shale oil in the Jimusaer sag.

At present, the identification standard of “sweet spot” of continental shale oil in Jimusaer is not perfect, and the problems such as large reservoir depth, thin drilling target layer, and high sand adding strength result in a low drilling encounter rate and low fracturing efficiency, and it is difficult to reduce the cost and increase the efficiency of exploration and development of shale oil. Therefore, how to effectively identify the “sweet spot” is a key problem. Free oil is the main producing part of the volume fracturing development of horizontal wells, and its content represents the material basis for exploitation. Conventional parameters [such as total organic carbon (TOC), *S*_1_, OSI (*S*_1_/TOC × 100%), etc.] used to characterize the oil-bearing property in shale can only indirectly reflect the free oil content. The application of two-dimensional NMR (nuclear magnetic resonance) and QGF (quantitative grain fluorescence) technology in the evaluation of the free oil content provides a way for unconventional shale oil development.^14^ The Lucaogou Fm in the study area is characterized by variable lithology, diverse mineral composition, and frequent interbedded distribution of the source rock and reservoir, 2D NMR can solve the above problems by distinguishing the solid organic matter and oil signal. Combined with fluorescence thin section examination, fluid mobility can be identified, which provides a strong basis for sweet spot identification.

Based on this, P_2_l_1_^2^ of the typical well (well Ji-X) in the western sag was taken as the research object. Through thin section identification and X-ray diffraction (XRD) of whole rock and clay minerals, the mineralogical and petrological characteristics were analyzed and the main lithologies were determined. The geochemical characteristics of source rocks were analyzed through TOC, pyrolysis, maceral identification, and the differences in petroleum generation potential between the eastern and western source rocks were compared; the micro- and nanopore characteristics and pore structural characteristics of the shale oil reservoir were studied by high-pressure mercury injection and scanning electron microscopy (SEM). The oil contents were analyzed in detail by fluorescence thin section, 2D NMR, fluorescence QGF, and fluid saturation tests. On the basis of the above, the vertical distribution characteristics of the oil content and its main controlling geological factors have been systematically evaluated, and the accumulation mechanism of shale oil is summarized. The research results should be of great significance for shale oil exploration in the Junggar Basin and potentially other regions in the world.

## 2 Geological Setting

**Figure 1:**
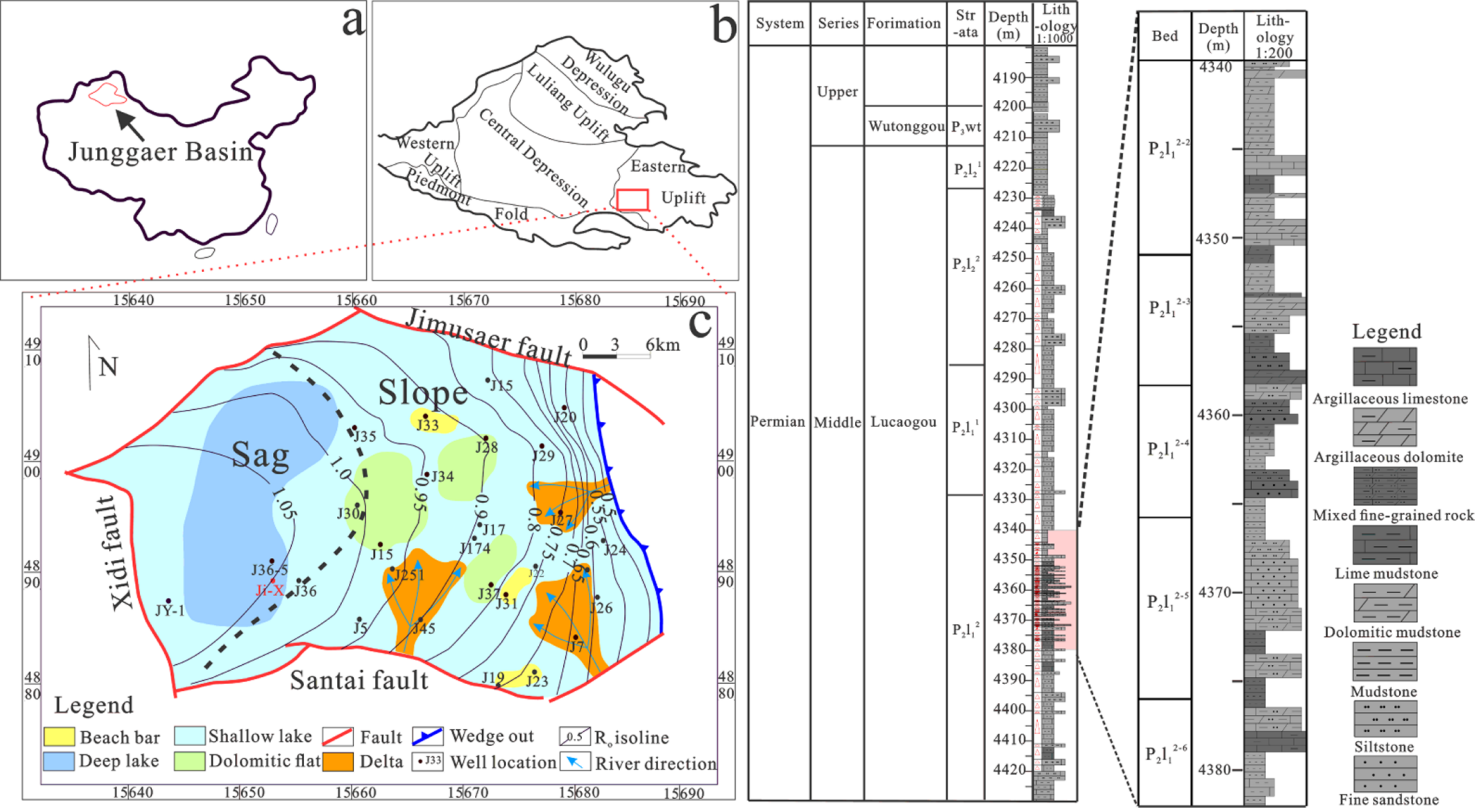
(a) Location of Junggar basin, (b) location of Jimusaer depression, and (c) typical stratigraphic column of Jimusaer depression.

The Jimusaer depression is located in the southeast margin of the eastern Junggar basin and is surrounded by the Qitai uplift to the east, the Santai fault to the south, the Xidi fault to the west, and the Jimusaer fault to the north. The tectonic unit area is about 1278 km^2^, which is a half graben with high in the east and low in the west (Figure 1). The western part is the sag area and the eastern part is the slope area. The slope area is considered to be the major development area of unconventional oil reservoirs. Several wells have obtained an industrial oil flow.^15,16^ Recently, with the increase of the exploration degree, it is found that the Lucaogou Fm in the western sag shows great potential for shale oil development.

During the deposition of the Lucaogou Fm in Jimusaer sag, the main sedimentary environments include delta, sandy shoal, carbonate shoal, dolomitic flat in a shallow water environment, and deep lake in a deep water environment.^16,17^ The thickness of the Lucaogou Fm is between 25 and 300 m, with an average of 200 m. The burial depth is between 800 and 4500 m, with an average of 3570 m. The burial depth gradually increases from east to west. The Lucaogou Fm is divided into two members (P_2_l_1_ and P_2_l_2_) from bottom to top, with the development of the upper and lower sweet spots. The lower sweet spot is located in part 2 of Mbr 1 of the Lucaogou Fm (P_2_l_1_^2^). The terrigenous clastic supply is sufficient during the development of this section^18^ and consists mainly of interbedded sandstone, mudstone, and dolomite (Figure 1).^19^ There is frequent interbedding between organic-rich mudstone and reservoir in the sweet spot area, and the boundary between the source rock and reservoir is not clear, showing a large area of interbedded superposition distribution and relatively stable lateral distribution. It is a typical source and reservoir symbiotic shale oil reservoir.

## 3 Samples and Methods

A total of 43 samples were taken from P_2_l_1_^2^ in well Ji-X with a total thickness of 43 m, and the sampling interval was one sample per meter. The analyses included mineralogy and petrology, source rock geochemistry, pore properties, and oil content test.

### 3.1 Mineralogy and Petrology

All 43 samples were analyzed by a Bruker D8 Discover X-ray diffractometer according to the Chinese industry standard (SY/T 5163-2018).^20^ Samples were milled and passed through a 200 mesh sieve. The suspension method was used to determine the relative clay content. Clay minerals with a diameter of less than 2 μm were extracted, and a directional film was produced that included natural air-dried pieces, ethylene glycol pieces, and pieces heated at 550 °C. The content of the clay mineral was calculated by the diffraction peak intensity contrast method and adiabatic equations.^21^

### 3.2 Bulk Geochemistry

All 43 samples were tested for bulk geochemistry, including the TOC and Rock-Eval pyrolysis. In order to determine the total oil content, 19 samples were selected for TOC and Rock-Eval pyrolysis tests after solvent extraction. The TOC content was determined according to the Chinese standard (GB/T 19145-2003).^22^ First, dilute hydrochloric acid was used to dissolve carbonates, and then, the TOC content was determined using a CS-230 analyzer, with an infrared detector to quantify the CO_2_ formed from the combustion of the organic carbon. A Rock-Eval 6 was used to determine *S*_1_, *S*_2_, and *T*_max_ (the temperature of maximum pyrolysis *S*_2_ yield) according to the Chinese standard (GB/T 18602-2012).^23^*S*_1_ was measured at 300 °C for 3 min, and *S*_2_ was determined after the temperature was programmed to 550 °C.

The maceral compositions of 21 samples were determined according to the Chinese industry standard (SY/T 6414-2014).^24^ In this study, the percentage of a single maceral was determined using the same polishing blocks cut perpendicular to the stratified surface. Maceral analysis was performed using a Leica MPV microscope with incident white light and blue light, 50 times objective lens, and oil immersion method. At least 1000 points are calculated for each polished block.^25^

### 3.3 Pore Properties

The pore structure analysis was performed on 22 samples using a PoreMaster-60 type automatic mercury pressure apparatus. The contact angle between the mercury and the sample surface was 140°, the surface tension of mercury was 480 dyn/cm, and the pore diameter range was larger than 3.0 nm. Field-emission SEM of FEI quanta 450 is used to classify the reservoir space types.

High-pressure mercury injection experiments can effectively describe the pore size and pore-throat combination relationship, which are the key factors to control the permeability of the shale oil reservoir.^10^ Pores have self-similarity within a certain scale range, and pores of different scales have different fractal dimensions. Therefore, fractal theory can be used to guide the division of pore boundaries. The calculation of the pore fractal dimension is as follows:^21^

The relationship between the pore radius and the experimental pressure is as follows
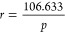
1-1

According to the fractal definition, when measuring the pore volume (*V*_m_) with a cylinder with a height of *L* and a radius of *R*, the following relationship exists between *V*_m_ and *D*
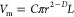
1-2
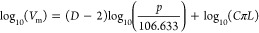
1-3
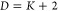
1-4Note: *D* is the fractal dimension, *r* is the pore radius, nm; *p* is the experimental pressure, psi; *C* is the proportionality constant; and *K* is the slope of *V*_m_ and log_10_(*p*/106.633).

### 3.4 Oil Content

Oil content tests included 2D NMR (43 samples), fluorescence QGF test (43 samples), fluid saturation test (22 samples), and crude oil group composition analysis (33 samples).^13,26^

A NMR instrument with an experimental frequency of 23 MHz was used to quantitatively divide the organic matter abundance and light hydrocarbon fluid in samples. The advantage of 2D NMR lies in the ability to divide the *T*_1_–*T*_2_ map into four parts, which are solid organic (*T*_1_ > 10 ms and *T*_2_ < 0.2 ms), light crude oil (*T*_2_ > 0.2 ms and *T*_1_ > 10 ms), hydroxyl-rich compounds (*T*_2_ < 0.2 ms and *T*_1_ < 10 ms), and water (*T*_1_ < 10 and 1 ms > *T*_2_ > 0.2 ms).^14,27^

The fluorescent test was carried out according to the Chinese industry standard (SY/T 7309-2016).^28^ First, the sample was crushed to a size of 80–100 mesh, weighed 2 g, and then added into 20 mL of dichloromethane solution, and the extraction solution was obtained by ultrasonic vibration. QGF-E represents the fluorescence characteristics of the hydrocarbon extraction solution adsorbed on the surface of reservoir particles. λ_max_ and QGF-E intensity are the two most important parameters. The former reflects the composition and density of crude oil, and the latter mainly represents the oil saturation.^14^ TSF (total scanning fluorescence) technology can be used to detect the three-dimensional fluorescence spectrum of the QGF-E solution, which can reflect the hydrocarbon composition information more comprehensively. TSF and QGF-E technology can be used to judge the hydrocarbon properties and conduct fine oil source correlation.^29^ Oil characteristics can be determined by MAX-E_X_, R_1_, TSF-MAX, MAX-E_M_ (under 270 nm excitation light, the ratio of *I*_360nm_ to *I*_320nm_), and *R*_2_ (under 260 nm excitation light, the ratio of *I*_360nm_ to *I*_320nm_).^30^ The lower the aromatic content and TSF-MAX value, the higher the API and the lighter the oil quality. The smaller *R*_1_ and *R*_2_, the higher the maturity of crude oil, and the lower the density of crude oil.

The fluid saturation is determined by the distillation extraction method. Toluene is used to extract oil in the oil–water core, dry it, and get the oil saturation according to the weight of the core before and after extraction. At the same time, the water in the core is evaporated out by the extraction process, condensed, and gathered in the scale tube of the water catcher. After the water in the tube does not increase, the volume of the water is read out, and then the water saturation is calculated.

Gas chromatography is used to analyze the bulk composition of the oil according to the Chinese industry standard (SY/T 5119-2016).^31^ First, *n*-hexane was used to filter the asphaltene solution in the rock soluble organic matter or crude oil. The filtrate was eluted with *n*-hexane, dichloromethane and *n*-hexane (volume ratio 2:1), anhydrous ethanol, and chloroform to obtain saturated hydrocarbon, aromatic hydrocarbon, and nonhydrocarbon in turn. Then, the solvent of each component is volatilized until a constant weight is achieved, and the content of each fraction is normalized to 100%.

## 4 Results

### 4.1 Mineralogy

**Figure 2:**
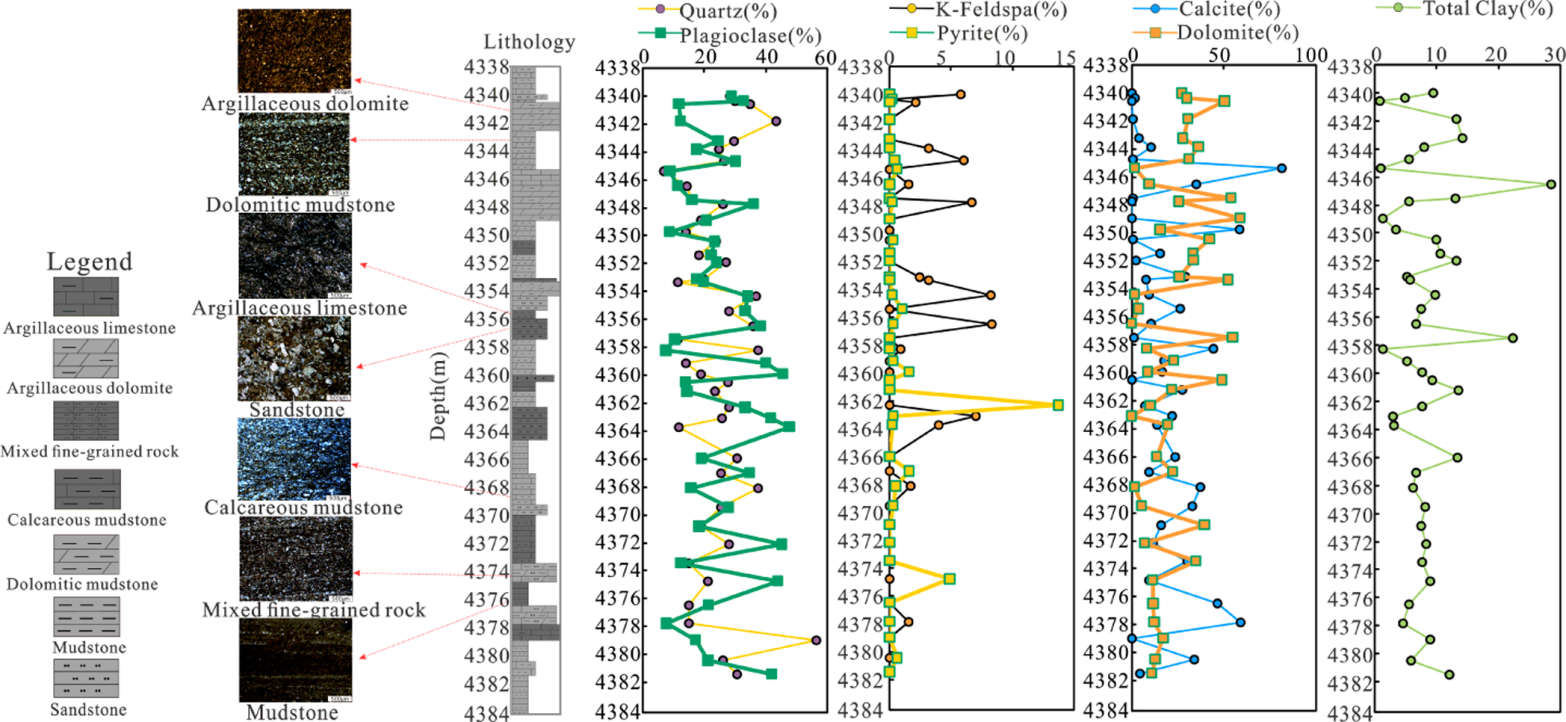
Comprehensive lithology profile of P_2_l_1_^2^ in well Ji-X.

On the basis of the systematic observation of the core, combined with the thin section and XRD analysis results (Table 1), the lithologic characteristics and variation of P_2_l_1_^2^ in well Ji-X were analyzed. The results show that this succession is a set of mixed fine-grained sediments of sandstone, mudstone, and carbonate under the combined action of mechanical deposition, chemical deposition, and biological deposition in a saline lake environment, with thin lithology thickness and frequent interbedding (Figure 1). The rock type is mainly the transitional rock from dolomite to siltstone, which is generally divided into sandstone, mudstone, dolomite, and a small number of other rock types. It can be divided into seven subgroups: dolomitic mudstone, calcareous mudstone, mudstone, mixed fine-grained rock, argillaceous limestone, sandstone, and argillaceous dolomite (Figure 2).

**Table 1:** XRD Results of P_2_l_1_^2^ of Well Ji-X^a^

		whole rock mineralogy (wt %)							clay mineralogy (wt %)					
no.	depth (m)	Q	K-F	P	Cal	Dol andFe-Dol	Py	TC	S	I	K	C	I/S	C/S
1	4340	28.4	5.8	28.9	0.2	27.3	0	9.4	94	6	0	0	0	0
2	4340.35	30	0	33	1.8	30.1	0.2	4.9	12	33	6	18	31	0
3	4340.56	34.8	2.1	11.8	0.2	50.4	0	0.8	7	24	26	24	19	0
4	4341.8	43.3	0	12.4	0.6	30.5	0	13.2	67	14	0	10	0	0
5	4343.22	29.7	0	24.6	3.9	27.7	0	14.1	75	17	0	8	0	0
6	4343.84	24.7	3.2	17.6	10.2	36.3	0	8	26	44	0	16	14	0
7	4344.7	26.3	6	30.4	0.4	31	0.4	5.5	26	43	10	11	10	0
8	4345.36	6.9	0	8.9	81.1	1.5	0.6	0.9	4	48	0	33	15	0
9	4346.47	14.4	1.5	11.3	34.9	9.5	0	28.5	13	46	0	26	15	0
10	4347.47	16.1	0	16.1	0.7	54	0	13	38	40	7	12	3	0
11	4347.74	26	6.7	36.1	0	25.5	0.2	5.5	0	46	0	23	31	0
12	4348.93	18.9	0	20.8	0	59	0	1.3	3	62	0	20	15	0
13	4349.74	13.9	0	8.8	58.5	15.5		3.3	16	43	6	17	18	0
14	4350.43	23.7	0	23.4	0.5	42.2	0.3	10	100	0	0	0	0	0
15	4351.42	18.3	0	22.3	15.5	33.3	0	10.6	32	43	0	14	11	0
16	4351.94	27.1	0	24	2.2	33.5	0	13.2	100	0	0	0	0	0
17	4353.11	20	2.4	17.6	29	25.9	0	5.1	25	58	0	10	7	0
18	4353.33	11.3	3.2	19.8	7.5	52.4	0	5.7	31	29	0	27	13	0
19	4354.38	36.8	8.2	34.3	9.4	1.5	0.2	9.7	86	5	0	9	0	0
20	4355.4	28.1	0	33.4	26.2	3.9	1	7.4	0	39	0	13	48	0
21	4356.47	36	8.3	38.5	10.3	0	0.3	6.6	0	30	0	10	5	55
22	4357.45	11.4	0	10.5	1.1	54.7	0	22.4	39	53	0	0	8	0
23	4358.24	37.7	0.9	7.6	44.2	8.3	0	1.3	7	51	0	15	27	0
24	4359.12	14.1	0	40.2	17.3	23	0.3	5.1	29	64	3	4	0	0
25	4359.93	18.9	0	45.8	16.4	8.8	1.6	7.7	79	1	0	20	0	0
26	4360.51	27.8	0	13.9	0	49	0	9.3	34	51	0	15	0	0
27	4361.19	23.4	0	14.4	27.2	21.6	0	13.5	18	52	0	11	19	0
28	4362.32	28	0	33.4	7.3	10	13.8	7.6	9	51	9	10	21	0
29	4363.08	25.9	7	41.7	21.7	0	0.3	2.9	3	69	0	0	11	17
30	4363.71	11.7	4	47.8	13.6	19.6	0.2	3.1	77	9	0	14	0	0
31	4365.99	30.8	0	19.1	23.5	13.4	0	13.3	33	30	4	10	23	0
32	4367.04	25.3	0	34.9	9.1	22.4	1.6	6.6	37	31	5	11	16	0
33	4368.11	37.5	1.7	15.7	37.1	1.4	0.5	6.2	8	61	5	3	23	0
34	4369.5	25.5	0	28	32.8	5.3	0.3	8.1	100	0	0	0	0	0
35	4370.87	18.8	0	18.4	15.9	39.5	0	7.5	36	24	14	26	0	0
36	4372.15	28	0	45.3	11.3	7.2	0	8.3	64	19	5	12	0	0
37	4373.46	15.1	0	12.5	30	34.8	0	7.6	30	41	13	16	0	0
38	4374.79	21.3	0	44	9.3	11.5	4.9	9	92	5	0	3	0	0
39	4376.48	15	0	21.5	46.5	11.6	0	5.5	31	50	0	19	0	0
40	4377.81	15	1.5	7.8	59.1	12.2	0	4.5	45	32	0	9	14	0
41	4378.98	56.6	0	17.3	0	17.2	0	8.9	89	2	0	9	0	0
42	4380.46	26.1	0	21.3	33.6	12.7	0.6	5.8	22	68	0	8	2	0
43	4381.45	30.8	0	42.1	4.2	10.8	0	12	92	4	0	4	0	0

a Q: quartz; F: feldspar; P: plagioclase; Cal: calcite; Dol: dolomite; Py: pyrite; TC: total clay; I: illite; S: smectite; K: kaolinite; C: chlorite; I/S: illite/smectite mixed layer mineral; and C/S: chlorite/smectite mixed layer mineral.

The mineral components include quartz (6.9–56.6%, avg. 24.64%), potash feldspar (0.9–8.3%, avg. 1.45%), plagioclase (7.6–47.8%, avg. 24.59%), calcite (0.2–81.1%, avg. 17.54%), dolomite (0.5–59%, avg. 22.93%), siderite (0.5–0.7%, avg. 0.03), pyrite (0.2–13.8%, avg. 0.63%), and clay minerals (0.8–28.5%, avg. 8.21%) (Table 1).

**Figure 3:**
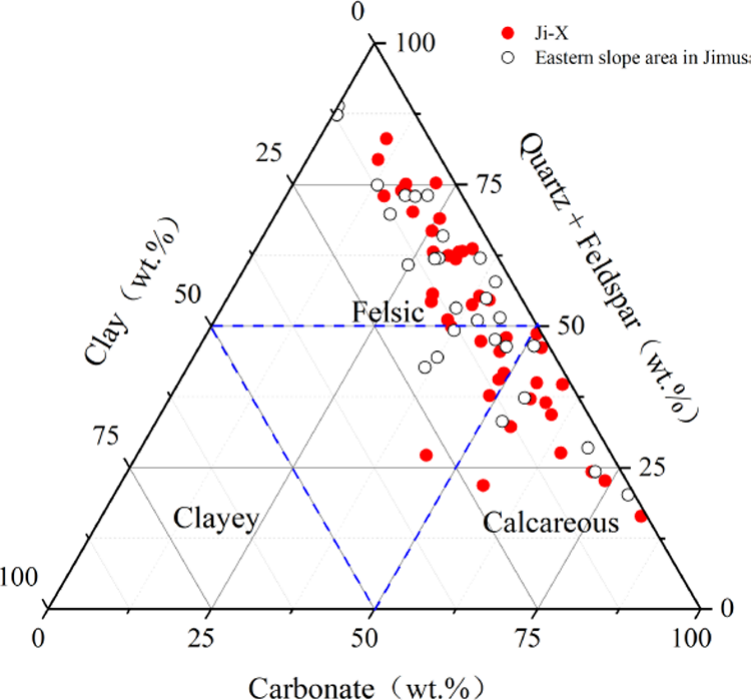
Ternary diagram of the mineral composition.

The mineral composition is complex with strong heterogeneity and is dominated by felsic fine-grained rocks (Figure 3), which is similar to the mineral composition of the slope area.^32^ Clay minerals are mainly smectite (0–100%, avg. 40.21%) and illite (0–69%, avg. 33.44%), and other minerals are relatively minor, including I/S mixed layer mineral, kaolinite, and chlorite. The higher content of smectite is closely related to alkaline volcanic materials.^33^

### 4.2 Geochemical Characteristics

The TOC content covers a range from 0.42 to 19.12% (averaging 3.89%) (TOC content more than 2.0% account for 74% of the total), which is higher than that in the slope area (averaging 3.49%).^13^ According to the *R*_o_ value calculated by the *T*_max_ value, the source rock is generally mature, reaching the oil window (Table 2).

**Table 2:** TOC Content and Rock-Eval Pyrolysis Data for P_2_l_1_^2^ of Well Ji-X^a^

number	TOC (wt %)	*T*_max_ (°C)	*S*_1_ (mg/g)	*S*_2_ (mg/g)	HI (mg/g·TOC)	calc. *R*_o_(%)	number	TOC (wt %)	*T*_max_ (°C)	*S*_1_ (mg/g)	*S*_2_ (mg/g)	HI (mg/g·TOC)	calc. *R*_o_ (%)	number	TOC (wt %)	*T*_max_ (°C)	*S*_1_ (mg/g)	*S*_2_ (mg/g)	HI (mg/g·TOC)	calc. *R*_o_ (%)
1	1.10	445	0.49	4.32	392.73	0.85	23	4.79	449	1.26	35.43	739.67	0.92	4*	2.19	436	1.44	9.14	417.35	0.68
2	4.73	433	12.57	29.20	617.34	0.63	24	2.15	446	0.70	12.99	604.19	0.86	6*	4.11	443	1.30	20.00	486.62	0.81
3	4.28	433	12.72	30.14	704.21	0.63	25	0.42	444	0.13	1.54	366.67	0.83	10*	4.43	441	0.28	20.86	470.88	0.77
4	3.73	436	7.29	18.98	508.85	0.68	26	2.46	441	0.70	10.74	436.59	0.77	12*	1.25	440	0.24	4.89	391.20	0.7
5	1.95	441	1.94	6.98	357.95	0.77	27	10.27	447	1.35	69.22	674.00	0.88	14*	1.00	441	0.12	2.76	276.00	0.77
6	4.48	440	4.00	23.04	514.29	0.76	28	4.61	439	5.51	22.50	488.07	0.74	16*	1.29	440	0.19	4.53	351.16	0.7
7	2.90	438	5.77	14.39	496.21	0.72	29	2.88	439	10.75	17.04	591.67	0.74	18*	1.22	436	0.66	4.97	407.38	0.68
8	5.86	440	7.95	31.66	540.27	0.76	30	1.55	430	5.92	7.77	501.29	0.58	20*	0.62	435	0.35	1.64	264.52	0.67
9	3.71	443	3.90	27.49	740.97	0.81	31	10.67	448	2.69	69.23	648.83	0.90	22*	0.42	441	0.06	0.65	154.76	0.77
10	4.72	446	0.92	26.15	554.03	0.86	32	2.72	428	4.55	13.75	505.51	0.54	24*	0.69	445	0.04	2.83	410.14	0.85
11	2.40	432	7.67	12.11	504.58	0.61	33	7.11	449	1.44	53.47	752.04	0.92	26*	1.29	440	0.06	4.39	340.31	0.76
12	2.30	441	7.37	12.60	547.83	0.77	34	0.88	439	1.07	3.45	392.05	0.74	30*	0.46	438	0.05	1.66	360.87	0.72
13	7.98	445	1.22	52.70	660.40	0.85	35	4.87	452	1.03	42.64	875.56	0.97	32*	1.24	431.	0.12	4.50	362.90	0.59
14	1.04	441	0.68	3.66	351.92	0.77	36	1.57	444	2.26	8.63	549.68	0.83	34*	0.70	441	0.14	1.85	264.29	0.77
15	6.08	447	3.39	34.57	568.59	0.88	37	4.55	446	1.21	31.19	685.49	0.86	36*	1.02	447	0.27	4.80	470.59	0.88
16	1.66	435	1.00	6.55	394.58	0.67	38	3.69	452	1.18	28.21	764.50	0.97	38*	3.16	450	0.24	23.28	736.71	0.94
17	4.98	447	3.07	32.72	657.03	0.88	39	3.10	448	0.65	20.58	663.87	0.90	40*	2.24	448	0.09	13.69	611.16	0.9
18	2.03	433	4.10	9.19	452.71	0.63	40	3.02	449	0.57	21.97	727.48	0.92	42*	18.07	453	0.19	129.36	715.88	0.99
19	0.49	439	0.59	1.41	287.76	0.74	41	1.27	445	0.57	5.81	457.48	0.85							
20	1.22	428	3.21	4.89	400.82	0.54	42	19.12	458	2.62	139.92	731.80	1.08							
21	2.49	429	8.12	13.04	523.69	0.56	43	4.93	450	0.87	39.74	806.09	0.94							
22	0.59	436	0.75	2.20	372.88	0.68	2*	1.23	440.00	0.15	4.42	359.35	0.76							

a * indicates the sample after solvent extraction; calc. *R*_o_ from *T*_max_ = 0.018 × *T*_max_ – 7.16.

**Figure 4:**
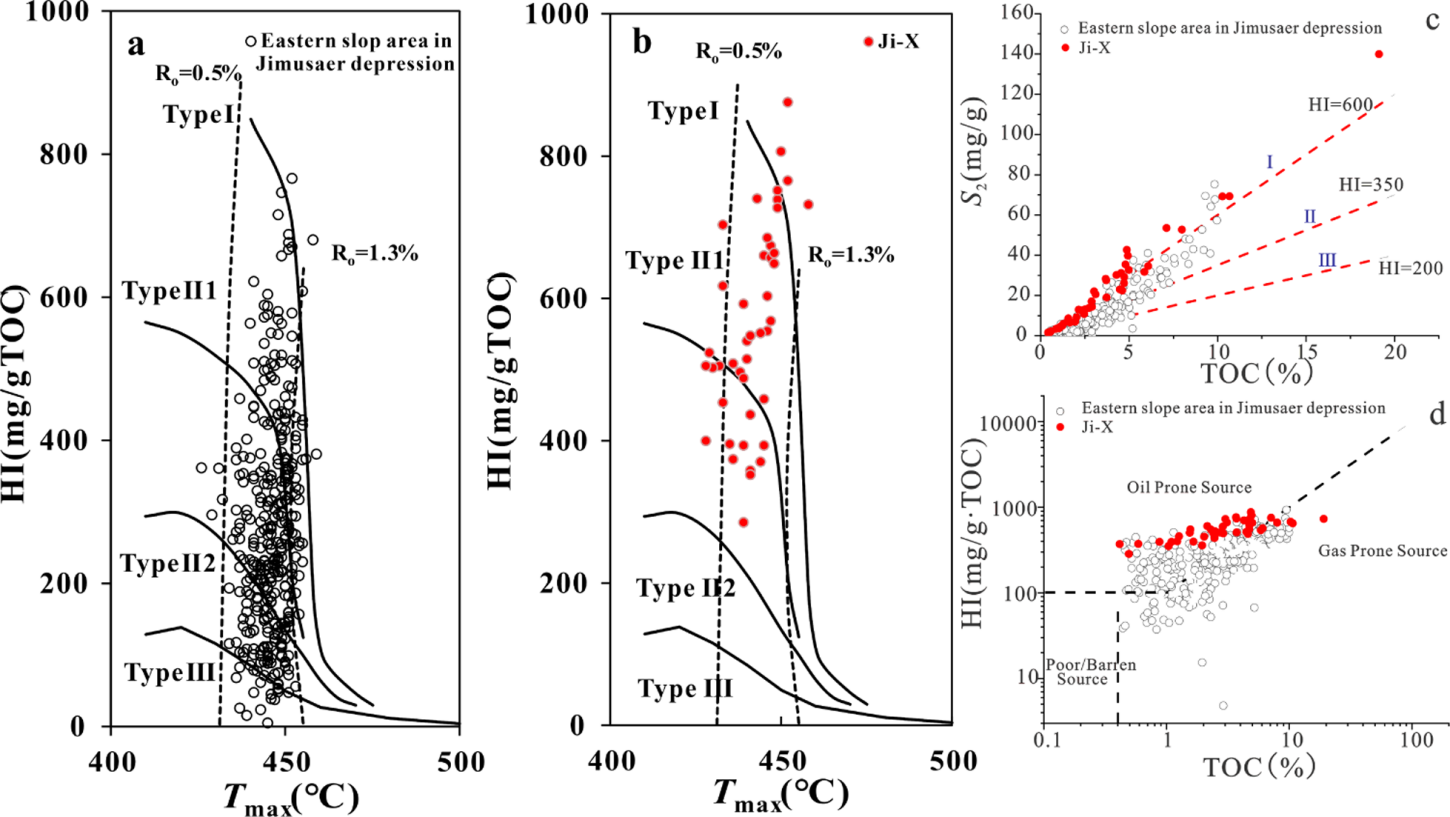
(a) Relationship between *T*_max_ and hydrogen index of the slope area in Jimusaer depression. (b) Relationship between *T*_max_ and hydrogen index of Ji-X well. (c) Correlation between S_2_ and TOC contents. (d) Correlation between HI and TOC contents.

The organic matter type of the Lucaogou Fm source rock in the whole exploration area of Jimusaer is mainly type II, and the organic matter type of P_2_l_1_^2^ in well Ji-X is the best (type I–II) (Figure 4a,b). There is a significant positive correlation between *S*_2_ and TOC, and type I accounts for the largest proportion (Figure 4c). In addition, the relationship between HI and TOC indicates that P_2_l_1_^2^ of well Ji-X in the western sag is the best source rock in the whole exploration area of Jimusaer (Figure 4d).

### 4.3 Pore Types and Pore Distribution Characteristics

#### 4.3.1 Pore Type

**Figure 5:**
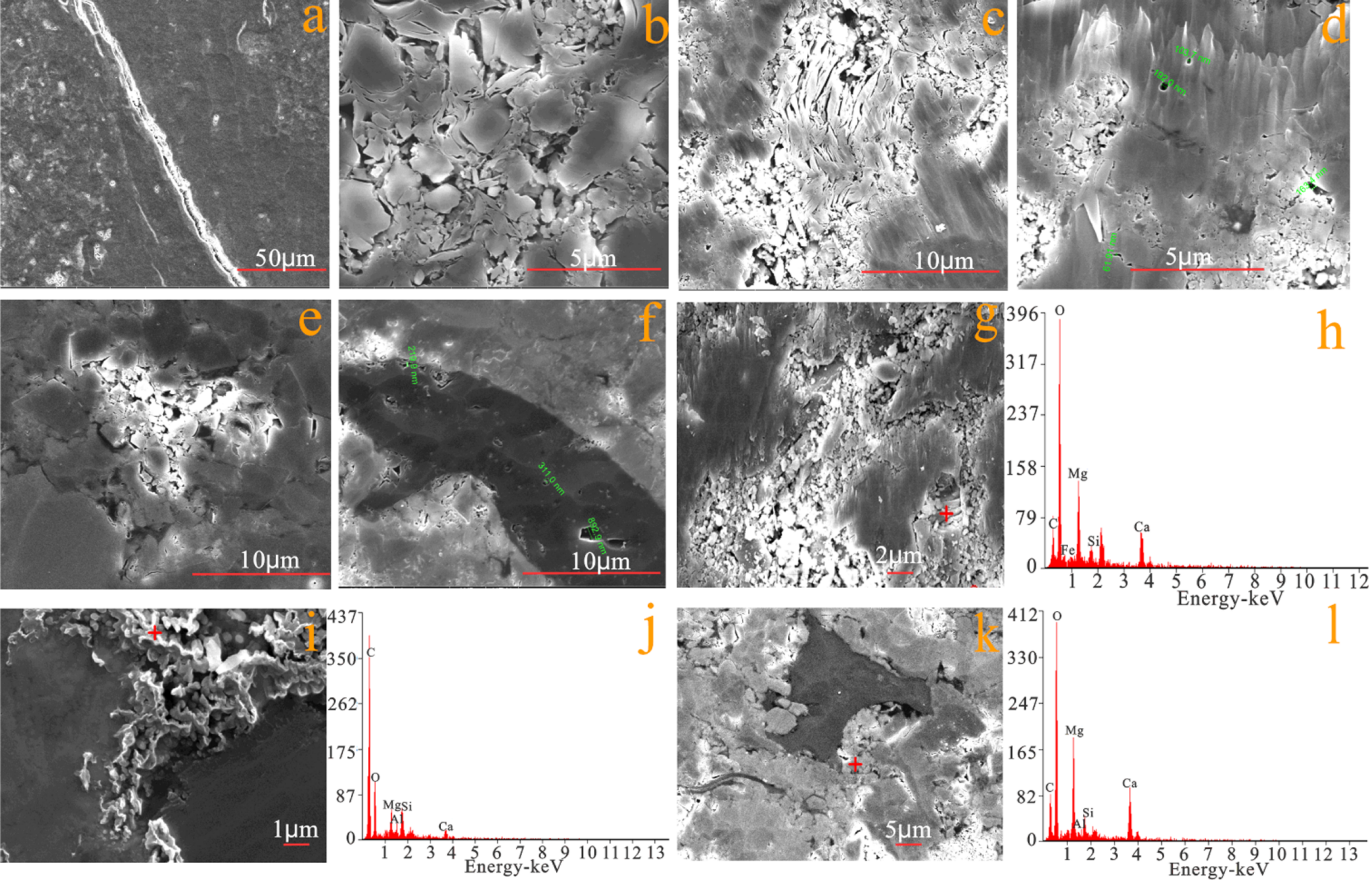
SEM diagram of the pore. (a) Microcrack, (b) residual intergranular pore, (c) intercrystalline pore of the clay mineral, (d)intragranular dissolved pore, (e) intergranular dissolved pore, (f) organic pore, (g) dissolved pore of dolomite of sample 33, (h) energy spectrum of sample 33, (i) dissolved pore of dolomite of sample 30, (j) energy spectrum of sample 30, (k) dissolved pore of dolomite of sample 6, and (l) energy spectrum of sample 6.

SEM shows that the pores in the samples can be divided into three categories: primary pore, secondary pore, and fracture, which can be divided into the residual intergranular pore, dissolved intergranular pore, dissolved intragranular pore, intercrystalline pore, organic pore, and microcrack (Figure 5a–f). The pore diameter range from small to large is the intergranular pore within clay particles, dissolved intragranular pore and intergranular pore, organic pore and dissolved intragranular pore, and organic pore and intergranular pore. The dissolved pores are mainly developed in dolomite (Figure 5g–l).

#### 4.3.2 Pore Structure Characteristics

**Figure 6:**
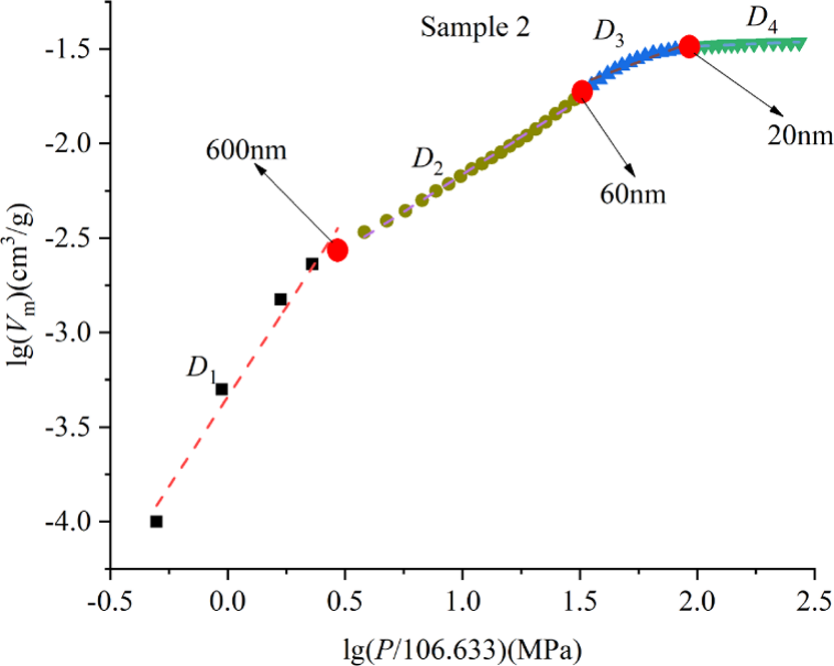
Relationship between lg(*V*_m_) and lg(*p*/106.633) of sample 2.

The results show that there are three turning points in the curve describing the relationship between lg(*V*_m_) and log_10_(*p*/106.633), indicating that the pore space of the samples has multifractal characteristics, and the pore diameters corresponding to the turning points are 600, 60, and 20 nm, respectively (Figure 6). Therefore, the curve can be divided into four linear segments, corresponding to the existence of four different pore structures: micropore (<20 nm), transitional pore (20–60 nm), mesopore (60–600 nm), and macropore (>600 nm).

The fractal dimension is often used to quantitatively characterize the complexity of the pore space. The fractal dimension of different pore sizes is related to reservoir physical properties, but it is difficult to fully represent the complexity of the whole sample. Therefore, taking the pore volume ratio of different pore sizes as the weight of the corresponding fractal dimension, the comprehensive fractal dimension is obtained by the weighted sum of the fractal dimensions of different pore sizes.^34^ The corresponding formula is as follows
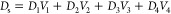
1-5Note: *V*_1_, macropore volume (>600 nm); *V*_2_, mesopore volume (60–600 nm); *V*_3_, transition pore volume (20–60 nm); and *V*_4_, micropore volume (<20 nm); *D*_1_, *D*_2_, *D*_3_, and *D*_4_ are fractal dimensions of macropore, mesopore, transition pore, and micropore, respectively.

The pore comprehensive fractal dimension of samples with different lithologies is between 2.49 and 4.58 (Table 3). Generally, the fractal dimension of porous solid media should be between 2.0 and 3.0. However, if the compression deformation of porous media occurs under high pressure, a fractal dimension greater than 3 may occur, but the result is still an effective index to characterize reservoir physical properties.

**Table 3:** Mercury Injection Data^a^

		pore volume (cm^3^/g)				fractal dimension				
no.	depth/m	*V*_1_	*V*_2_	*V*_3_	*V*_4_	*D*_1_	*D*_2_	*D*_3_	*D*_4_	*D*_s_
1	4340	4.95	14.84	25.27	54.95	2.19	2.70	2.70	2.77	2.71
2	4340.35	8.19	46.49	39.77	5.56	3.90	2.79	2.46	2.05	2.71
3	4340.56	0.00	91.96	7.04	1.01		4.80	2.05	2.01	4.58
6	4343.84	1.82	1.82	19.09	77.27	2.00	2.30	3.83	3.34	3.39
8	4345.36	1.11	46.80	44.01	8.08	2.41	3.26	2.46	2.06	2.80
11	4347.74	2.81	75.56	18.26	3.37	2.00	3.63	2.14	2.04	3.26
12	4348.93	0.55	80.87	16.39	2.19	2.00	3.65	2.10	2.02	3.35
14	4350.43	1.03	32.99	52.06	13.92	2	3.97	2.78	2.09	3.07
15	4351.42	9.46	5.86	22.97	61.71	3.12	2.21	2.86	2.83	2.83
17	4353.11	0.57	38.86	53.14	7.43	2	4.21	2.66	2.05	3.22
18	4353.33	3.12	88.39	8.50	0.00	2.22	3.46	2.06	2.00	3.30
19	4354.38	1.02	16.75	67.01	15.23	2.19	3.36	3.29	2.15	3.12
22	4357.45	0.80	50.40	29.60	19.20	2.00	3.14	2.37	2.17	2.72
23	4358.24	38.57	14.29	12.86	34.29	2.73	2.10	2.16	2.50	2.49
24	4359.12	8.86	15.19	22.78	53.16	2.68	2.40	2.45	2.69	2.59
25	4359.93	2.35	14.12	28.24	55.29	3.03	2.78	2.91	2.77	2.82
27	4361.19	7.46	24.63	20.15	47.76	3.17	2.48	2.46	2.64	2.60
29	4363.08	10.73	72.77	12.57	3.93	3.57	2.75	2.12	2.03	2.73
30	4363.71	6.09	62.17	26.96	4.78	2.64	3.16	2.28	2.02	2.83
35	4370.87	16.90	15.49	8.45	59.15	2.80	2.22	2.24	3.01	2.79
40	4377.81	0.00	6.45	19.35	74.19		2.51	3.34	3.07	3.09
43	4381.45	8.74	20.39	24.27	46.60	3.00	2.53	2.65	2.61	2.64

a *V*_1_, macropore volume content (>600 nm); *V*_2_, mesopore volume content (60–600 nm); *V*_3_, transition pore volume content (20–60 nm); *V*_4_, micropore volume content (<20 nm); *D*_1_, fractal dimension of the macropore; *D*_2_, fractal dimension of the mesopore; *D*_3_, fractal dimension of the transition pore; *D*_4_, fractal dimension of the micropore; and *D*_s_, comprehensive fractal dimension.

**Figure 7:**
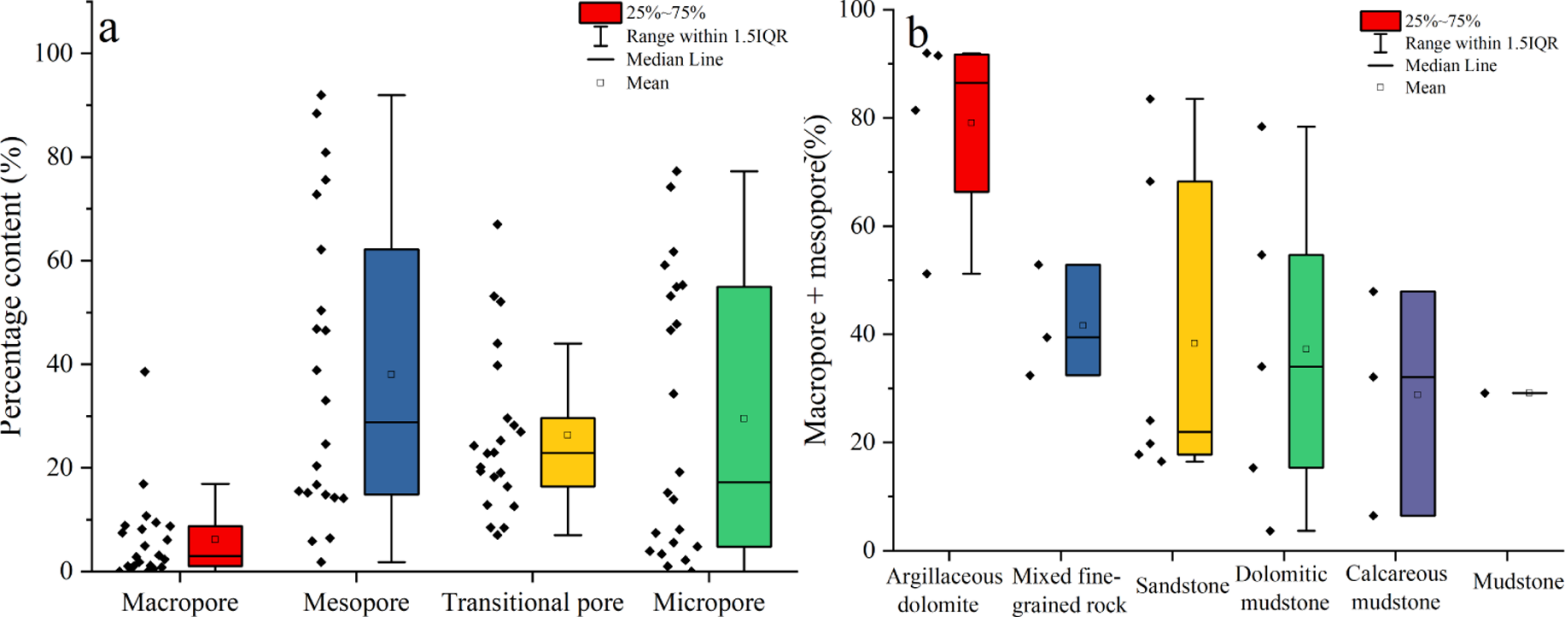
(a) Relative abundances of different pore types. (b) Content of macropores and mesopores in different lithologies.

The pore volume of different samples is dominated by mesopores, followed by transitional pores and micropores, with the macropores being the least abundant (Figure 7a). The content of the macropore and mesopore in argillaceous dolomite is the highest, indicating that it has relatively good reservoir physical properties, which may be related to the development of dissolved pores (Figures 5h–l and 7b).

### 4.4 Oil Content

#### 4.4.1 Oil Saturation

**Figure 8:**
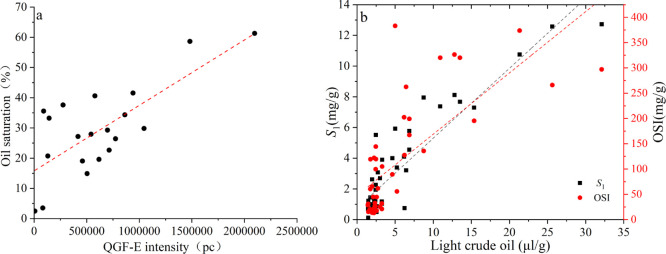
(a) Relationship between oil saturation and QGF-E intensity. (b) Relationship between light crude oil and *S*_1_, OSI.

The QGF-E intensity can be used to indicate oil saturation. Previous studies^14^ have shown that sandstone contains oil when the QGF-E intensity is higher than the 40 photometer count (pc). In this study, the normalized QGF-E intensity, in our samples, is as high as 2097291.5 pc, with an average normalized QGF-E intensity of 585727.4 pc, which is much higher than sandstone and Qingshankou Fm shale (average 124151.53 pc),^14^ indicating that it has good oil content (Table 4). The oil saturation and water saturation are determined using the fluid saturation test (Table 5). Oil saturation has a significant positive correlation with the QGF-E intensity, which confirms that our samples have a good oil content (Figure 8a). The oil saturation of P_2_l_1_^2^ ranges from 2.5 to 61.3%, with an average of 29.3%.

**Table 4:** Bulk QGF-E and TSF Parameters of the Samples from P_2_l_1_^2^ of Well Ji-X

	QGF-E		TSF						QGF-E		TSF				
number	λ_max_ (nm)	normalized QGF-E intensity (pc)	normalized TSF-MAX (pc)	MAX-E_X_ (nm)	MAX-E_M_ (nm)	*R*_1_ (nm)	*R*_2_ (nm)	number	λ_max_ (nm)	normalized QGF-E intensity (pc)	normalized TSF-MAX (pc)	MAX-E_X_(nm)	MAX-E_M_(nm)	*R*_1_ (nm)	*R*_2_ (nm)
1	373.0	27,4969.0	294,870.5	258	378	3.973	5.487	23	379.0	542,525.8	430,610.5	258	378	5.606	8.375
2	380.0	1,481,356.8	1,214,819.0	258	378	5.441	7.074	24	389.0	91,299.4	138,897.9	258	383	6.949	7.789
3	381.0	2,097,291.5	1,731,562.7	258	378	5.791	7.599	25	378.0	8042.0	8836.0	236	346	2.590	3.467
4	381.0	1,180,093.1	993,134.1	260	380	5.777	7.626	26	385.0	231,688.9	197,222.0	258	383	5.040	6.767
5	375.0	186,261.7	185,884.9	258	378	5.967	7.705	27	408.0	617,654.6	610,688.6	270	410	6.791	9.292
6	380.0	713,635.7	633,500.4	258	378	5.475	7.621	28	377.0	275,220.2	214,360.5	258	378	4.727	6.747
7	380.0	365,185.2	301,024.8	258	378	5.397	6.989	29	379.0	1,027,346.2	876,827.1	258	378	4.969	6.924
8	379.0	1,045,938.9	861,111.8	260	380	5.924	8.009	30	380.0	459,714.6	411,667.7	258	378	4.908	6.574
9	382.0	789,937.6	630,678.0	258	378	6.003	8.171	31	380.0	518,890.2	393,109.2	258	378	5.488	7.746
10	410.0	424,037.8	300,402.5	270	405	7.243	9.610	32	381.0	1,388,339.0	729,137.5	258	383	6.073	8.227
11	380.0	863,507.7	666,241.4	258	378	4.844	6.738	33	378.0	473,257.9	397,341.9	258	378	4.456	6.673
12	378.0	941,248.0	735,099.3	258	378	5.597	7.715	34	381.0	242,200.0	203,125.7	258	378	5.251	6.979
13	390.0	385,262.1	312,271.7	258	388	5.354	7.328	35	377.0	504,783.1	484,347.3	258	378	4.818	7.036
14	376.0	130,691.7	107,639.5	258	378	5.364	7.125	36	378.0	633,233.4	590,769.0	258	378	4.288	5.794
15	383.0	698,580.0	557,295.6	258	383	6.297	8.735	37	392.0	775,849.3	645,466.9	272	407	6.760	9.039
16	379.0	200,379.0	164,833.4	258	383	5.488	6.829	38	379.0	168,806.0	579,003.0	272	407	6.760	9.039
17	376.0	773,909.2	549,591.8	258	378	5.900	8.628	39	378.0	176,678.8	154,785.0	258	378	5.360	7.649
18	385.0	578,701.9	468,794.3	258	378	6.248	8.268	40	374.0	83,356.3	72,567.9	258	378	4.752	7.009
19	377.0	417,911.3	359,744.8	258	378	5.247	6.932	41	376.0	39,452.0	36,361.2	258	378	3.498	4.845
20	383.0	1,168,809.2	940,004.7	258	383	6.127	8.309	42	379.0	281,543.7	251,588.6	258	378	4.969	7.578
21	380.0	1,112,029.5	868,415.2	258	378	5.126	6.825	43	376.0	143,624.9	112,935.6	258	378	5.189	8.113
22	378.0	673,036.7	629,785.7	258	378	4.888	6.831								

**Table 5:** Fluid Saturation of P_2_l_1_^2^ of Well Ji-X

number	porosity (%)	water saturation (%)	oil saturation (%)	number	porosity (%)	water saturation (%)	oil saturation (%)	number	porosity (%)	water saturation (%)	oil saturation (%)
1	9.11	12.1	37.6	14	10.35	11.1	20.7	25	5.14	10.0	2.5
2	6.63	9.4	58.6	15	3.92	17.6	29.3	27	8.69	14.7	19.6
3	9.39	6.6	61.3	17	3.95	14.7	26.4	30	7.92	4.1	19.1
6	2.80	16.5	22.6	18	7.87	4.9	40.6	35	3.15	6.1	14.9
8	2.17	15.1	29.8	19	8.50	13.7	27.2	40	3.13	16.5	3.5
11	8.92	5.1	34.4	23	2.11	16.7	27.9	43	5.23	24.3	33.3
12	2.66	9.5	41.6	24	3.16	18.2	35.6				

The total fluid volume containing ^1^H was 226.7–1038.1 μL, with an average content of 562.2 μL. The amount of solid organic was 0–22.1 μL/g, with an average content of 4.3 μL/g, and the amounts of hydroxyl-rich compounds contained were 1.0–22.5 μL/g, with an average content of 7.7 μL/g. The water content in nanopores and fractures was 0–14.5 μL/g, with an average content of 3.5 μL/g, while the content of oil was 1.4–32.1 μL/g, with an average content of 5.8 μL/g (Table 7). The *T*_2_ value is positively correlated with fluid viscosity and fluidity. The higher the *T*_2_ value, the lower the fluid viscosity and the better the fluidity. The light oil (light hydrocarbons in oil) content has a significant positive correlation with *S*_1_ and OSI, which indicates that the higher the value, the stronger the fluid fluidity. This parameter can be used to divide the sweet section (Figure 8b).

**Table 6:** Crude Oil Group Components of P_2_l_1_^2^ of Well Ji-X

number	saturated hydrocarbon (%)	aromatic (%)	nonhydrocarbon (%)	asphaltene (%)	number	saturated hydrocarbon (%)	aromatic (%)	nonhydrocarbon (%)	asphaltene (%)	number	saturated hydrocarbon (%)	aromatic (%)	nonhydrocarbon (%)	asphaltene (%)
1	43.13	18.26	23.83	4.7	16	33.46	17.96	32.14	2.27	30	58.42	18.32	15.02	0.37
2	33.33	15.61	19.65	5.79	17	48.14	16.89	25.17	1.35	31	28.4	22.8	41.2	4.6
3	31.9	12.69	21.83	5.97	18	45.28	17.92	28.13	0.19	33	37.76	21.5	27.62	3.67
4	33.64	15.27	24.91	2.55	19	47.32	16.86	19.16	0.19	35	33.27	21.21	34.19	3.66
6	37.13	14.85	29.19	10.19	21	45.77	15.03	21.24	0.69	37	11.57	17.9	41.59	6.51
8	42.4	14.26	26.45	10.13	22	47.17	18.01	28.47	1.03	38	47.59	19.42	25.09	1.37
9	33.52	14.44	23.33	17.96	23	23.92	18.08	43.31	9.98	40	45.7	21.13	20.03	5.95
10	5.76	17.7	38.68	9.67	24	13.89	13.55	54.37	2.4	42	40.22	12.17	14.35	26.74
11	56.94	16.15	18.43	0.21	25	19.45	7.09	14.42	58.35	43	35.7	14.21	26.59	1.82
12	52.56	15.7	20.82	1.19	27	6.22	20.43	41.74	6.57					
13	6.89	15.8	45.38	15.46	28	41.62	16.96	23.89	4.82					
14	48.65	19.75	26.21	2.69	29	54.61	17.04	17.04	0.87					

**Table 7:** 2D NMR Data of P_2_l_1_^2^ of Well Ji-X

number	depth/m	sample quality (g)	content of all ^1^H (μL)	solid organic matter (μL/g)	hydroxyls (μL/g)	water (μL/g)	light crude oil (μL/g)	number	depth/m	sample quality (g)	content of all ^1^H (μL)	solid organic matter (μL/g)	hydroxyls (μL/g)	water (μL/g)	light crude oil (μL/g)
1	4340	30.527	541.889	0.000	11.145	4.030	2.575	23	4358.24	22.127	421.464	9.030	4.614	2.993	2.411
2	4340.35	31.226	900.295	0.000	1.644	1.568	25.620	24	4359.12	30.812	436.752	3.915	6.029	1.871	2.359
3	4340.56	28.333	956.28	0.000	1.035	0.611	32.105	25	4359.93	31.833	447.901	0.000	9.124	3.482	1.464
4	4341.8	22.278	849.436	5.746	13.941	3.077	15.366	26	4360.51	26.97	495.605	0.822	12.436	3.683	1.436
5	4343.22	30.382	622.852	1.550	12.997	3.504	2.449	27	4361.19	24.889	853.876	13.597	8.508	9.967	2.235
6	4343.84	28.091	737.124	7.398	10.420	3.777	4.645	28	4362.32	26.778	226.696	3.836	1.575	0.577	2.478
7	4344.7	30.481	505.426	2.340	6.789	0.585	6.868	29	4363.08	24.243	588.758	0.660	2.283	0.000	21.343
8	4345.36	29.651	531.989	7.317	1.503	0.372	8.749	30	4363.71	30.317	342.095	0.269	4.670	1.339	5.006
9	4346.47	25.751	546.571	10.994	4.992	1.934	3.305	31	4365.99	23.63	1016.492	12.025	13.447	14.526	3.019
10	4347.47	28.091	901.344	7.354	12.949	9.762	2.022	32	4367.04	22.561	355.452	2.416	5.314	1.160	6.865
11	4347.74	26.898	487.126	0.406	3.581	0.616	13.507	33	4368.11	20.721	438.346	10.227	7.397	1.795	1.736
12	4348.93	26.459	397.117	1.889	1.599	0.604	10.917	34	4369.5	26.075	432.013	0.000	11.093	3.227	2.247
13	4349.74	25.529	698.099	10.207	9.167	6.480	1.491	35	4370.87	20.725	577.869	7.013	9.614	7.986	3.270
14	4350.43	27.569	538.485	0.852	13.153	3.550	1.977	36	4372.15	24.006	301.825	1.773	6.906	1.433	2.461
15	4351.42	27.026	940.98	7.981	13.557	8.044	5.236	37	4373.46	21.747	531.575	4.491	8.891	8.719	2.343
16	4351.94	30.646	1038.081	0.333	22.545	9.237	1.758	38	4374.79	24.329	606.213	3.909	11.698	6.060	3.250
17	4353.11	27.54	330.946	3.509	4.165	1.606	2.738	39	4376.48	23.561	338.69	4.487	5.826	1.909	2.152
18	4353.33	28.348	382.677	1.901	4.017	1.399	6.181	40	4377.81	27.846	356.408	2.207	6.972	2.000	1.620
19	4354.38	29.875	493.04	0.284	10.694	3.765	1.760	41	4378.98	23.754	355.837	1.160	5.519	6.168	2.133
20	4355.4	27.921	328.557	0.232	4.358	0.731	6.447	42	4380.46	18.108	578.862	22.124	6.307	1.553	1.983
21	4356.47	42.823	814.445	0.635	5.305	0.286	12.793	43	4381.45	21.147	495.893	7.239	10.049	3.581	2.580
22	4357.45	30.211	431.174	1.721	5.411	0.903	6.236								

#### 4.4.2 Oil Quality Characteristics

The results from the TSF test show that the normalized TSF-MAX of our samples is between 8836 and 1731562.7 pc, with an average of 489450.1 pc. The MAX-EX ranges from 236 to 272, while MAX-EM falls between 346 and 410 nm. *R*_1_ is larger than 3 and *R*_2_ is greater than the overall *R*_1_ values. According to the standard proposed by Liu et al.:^30^ when *R*_1_ > 3 and the ratio of MAX-E_X_ to MAX-E_M_ is around 250/375 nm, it is medium heavy oil. The relevant parameters in the study area fall within the above range, reflecting the low maturity of crude oil and the overall heavy oil quality. The reasons are as follows: (1) in the strong reducing environment where the water body is salty, the organic matter such as algae is relatively rich, and the content of isoparaffins and naphthenes in the produced crude oil is relatively high;^35^ (2) the source rocks of Lucaogou Fm have a good organic matter type and early hydrocarbon generation, forming large-scale low mature shale oil, with a high content of gum and asphaltene (nonhydrocarbon + asphaltene distributed between 15.39 and 72.77%, with an average value of 34.65%) (Table 6). During the short-distance migration, the above components were not adsorbed by the formation but remained in the crude oil.^11^

## 5 Discussion

### 5.1 Main Controlling Factors of Oil Content

#### 5.1.1 Oil Content in the Dominant Lithofacies

**Figure 9:**
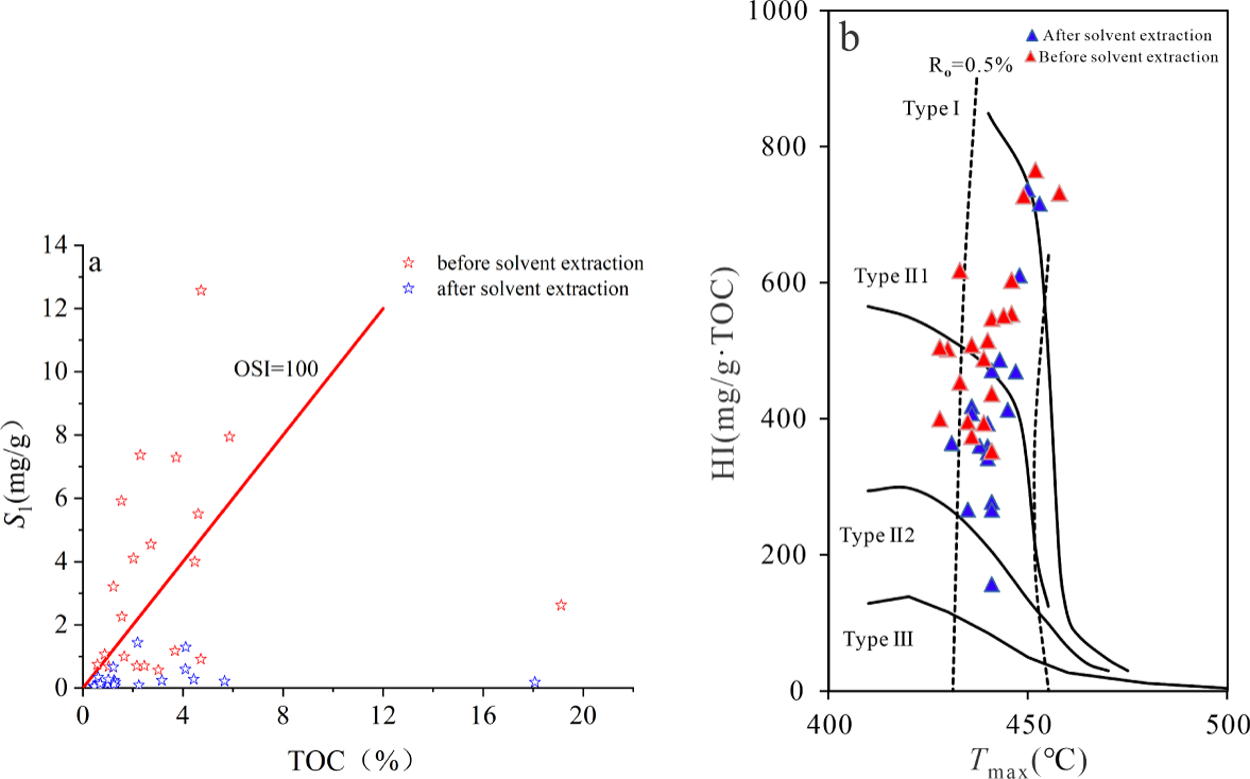
(a) Correlation between *S*_1_ and TOC content. (b) Correlation between HI and *T*_max_.

The HI did not change significantly before and after solvent extraction, but the *S*_1_ decreased significantly (Figure 9a), indicating that the core section contained abundant free oil, which was the same result as that obtained by 2D NMR (Figure 9b). Some low level residual *S*_1_ was observed after solvent extraction in some cases, but the values were low enough that the original *S*_1_ could be used to reflect the free oil content.

**Figure 10:**
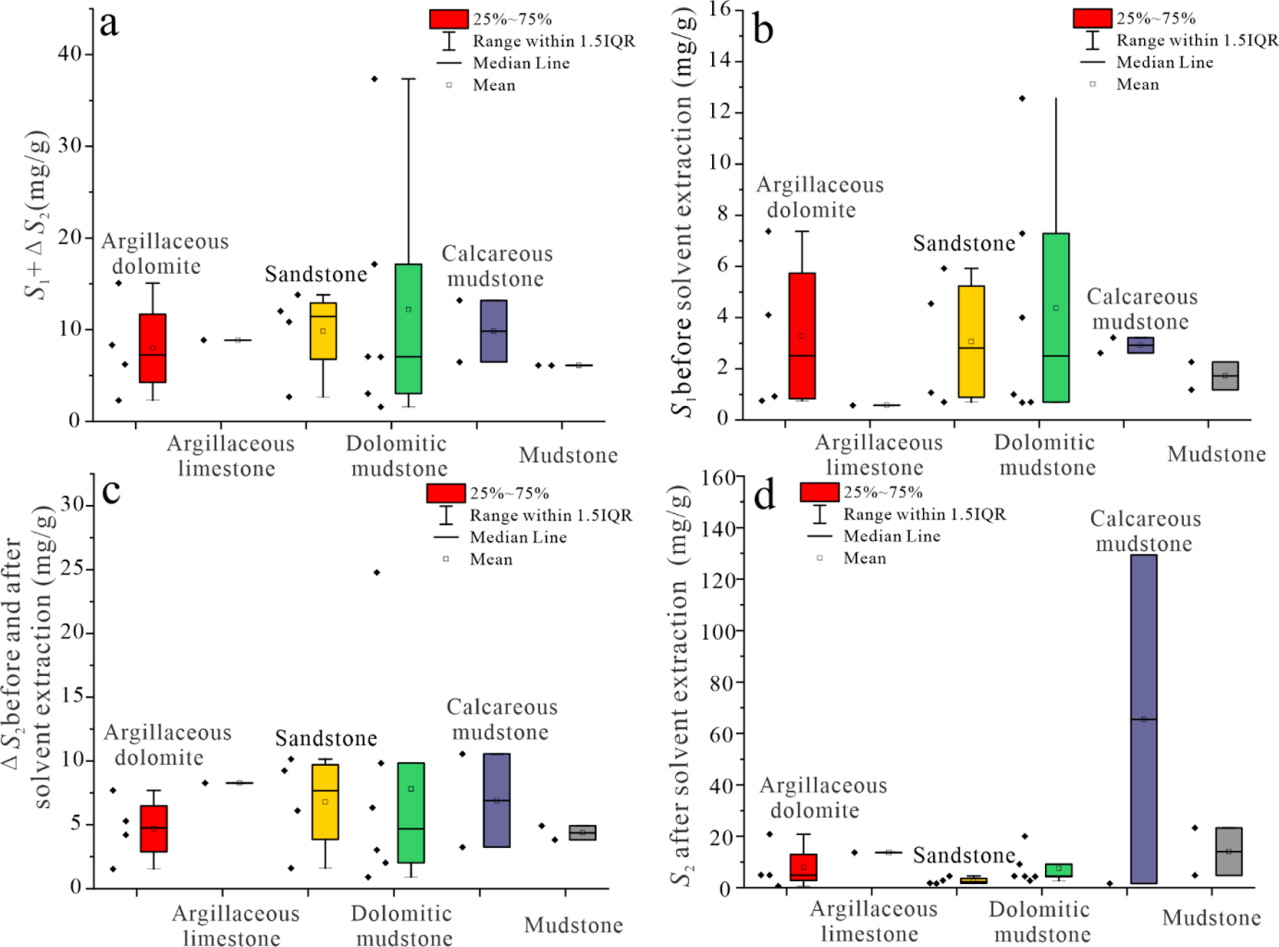
(a) Content of *S*_1_ + Δ*S*_2_ of different lithological lithologies. (b) Content of *S*_1_ before the solvent extraction of different lithological rocks. (c) Content of Δ*S*_2_ of different lithological rocks. (d) Content of *S*_2_ after the solvent extraction of different lithological rocks.

Δ*S*_2_ refers to the difference of *S*_2_ before and after solvent extraction. *S*_1_ + Δ*S*_2_ can reflect the total oil content indirectly, dolomitic mudstone, calcareous mudstone, and sandstone have a higher oil content (Figure 10a). From the original *S*_1_ before solvent extraction, the dominant lithofacies containing light oil are dolomitic mudstone, argillaceous dolomite, and sandstone (Figure 10b). According to Δ*S*_2_, the dominant lithofacies containing heavy oil are dolomitic mudstone, sandstone, and calcareous mudstone (Figure 10c). According to the *S*_2_ value after solvent extraction, the lithofacies of the best source rock are calcareous mudstone, which has the highest organic matter content but a high heavy component content (Figure 10d).

The average value of *S*_1_ before solvent extraction is less than Δ*S*_2_, indicating that the overall oil quality is relatively heavy. High light oil content and low organic matter content indicate the possibility of oil migration. Therefore, the favorable lithology for oil migration is dolomitic mudstone, argillaceous dolomite, and sandstone (Figure 10b,d).

#### 5.1.2 Influence of Reservoir Physical Properties on Oil Content

**Figure 11:**
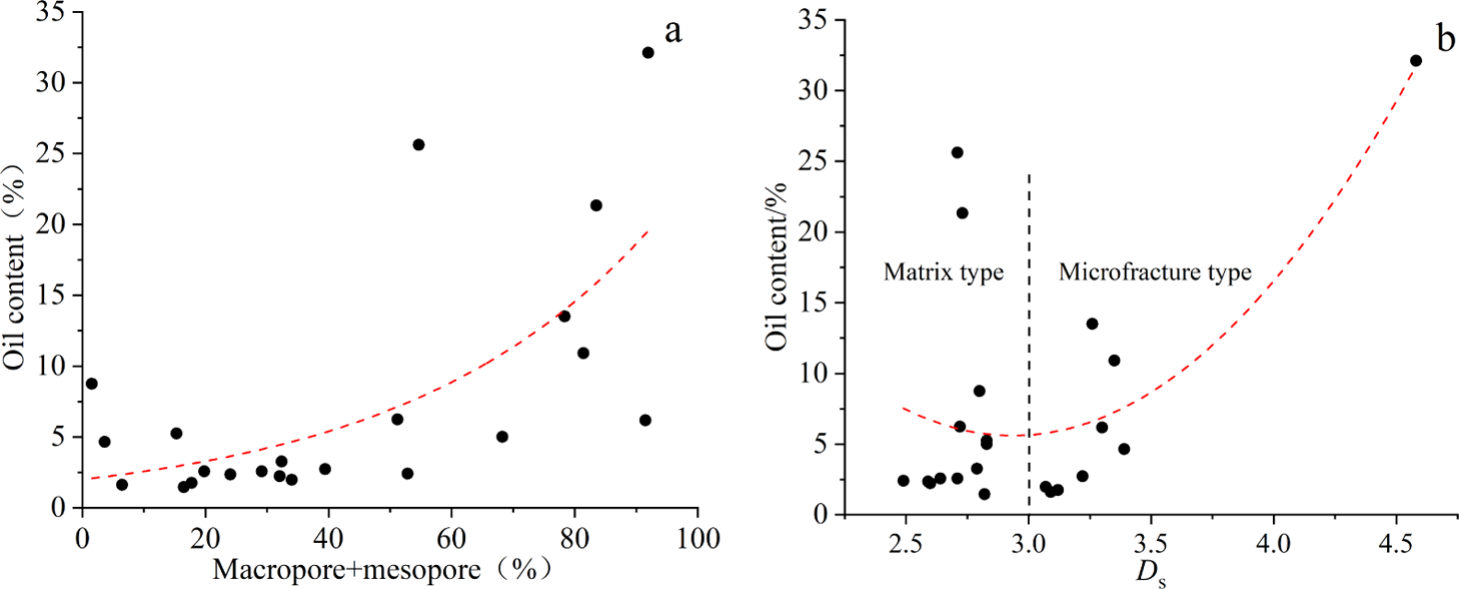
(a) Relationship between the oil content and the content of macropore + mesopore. (b) Relationship between oil content and *D*_s_.

The higher the content of macropores and mesopores, the higher the oil content (Figure 11a). The reason is related to the difference in wettability in different pore sizes. Previous studies have shown that the pore walls of the larger pore throats are generally covered by an oil film and have obvious characteristics of oil wetting, while the pore walls of the relatively small pore throats have no oil film development, which means that large pores are lipophilic (oil bearing) and small pores are hydrophilic (water bearing).^36^

In addition to the pore content, pore structure characteristics are also key to the oil content. The pore structure is often quantitatively characterized by the overall fractal dimension. The oil content changes in a parabolic manner with the increase in the fractal dimension, and there is a minimum point near *D*_s_ equal to 3 (Figure 11b). High pressure may cause the pores to break and the formation of microcracks, and this is the main cause of fractal dimensions greater than 3. Therefore, the pore types can be divided into the matrix type and microfracture type based on *D*_s_ equal to 3. For matrix-type samples, the main pore types are transition pores and micropores. The larger the fractal dimension, the stronger the heterogeneity of pores and the worse the physical properties of the reservoir, which is not conducive to oil accumulation. For microfracture-type samples, the main pore types are macropores and mesopores. The larger the content of these two pore types, the easier the microcracks can form under high pressures. In summary, the smaller the overall fractal dimension, the better the oil content, while the microfracture-type samples are the opposite.

### 5.2 Comprehensive Geological Characteristics

#### 5.2.1 Geological Characteristics of Sweet Spots

**Figure 12:**
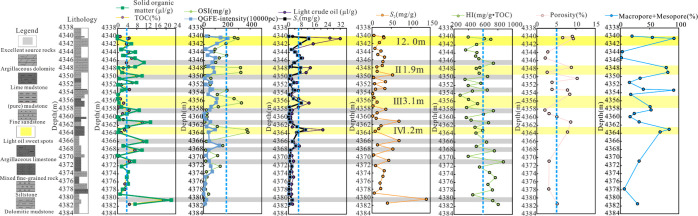
Division of “sweet spots” of P_2_l_1_^2^ of well Ji-X.

The variation in light oil content, OSI, and *S*_1_ with burial depth can directly reflect the vertical distribution of the sweet spots, and the three profiles are similar with increasing burial depth. The section is divided into two parts with a boundary at 4366 m, showing significantly different oil-bearing characteristics above and below. There are four (I, II, III, and IV) light oil sweet spots in the upper part (burial depth < 4366 m), which are characterized by high porosity, high content of macro- and mesopores, high OSI index (more than 200 mg/g·TOC), high *S*_1_, low organic matter content and low TOC content. There are excellent source rocks with high HI index (>600 mg/g·TOC) and high organic matter content (>4 μL/g) nearby, showing obvious thin source rock/reservoir interbedding. The effective thicknesses of the four sweet spots from shallow to deep are 2.0, 1.9, 3.1,and 1.2 m. There are source rocks with good petroleum generation potential in the lower part (burial depth > 4366 m). The HI of samples with high TOC is also high , but the light oil content is low and the physical properties are poor (Figure 12).

**Figure 13:**
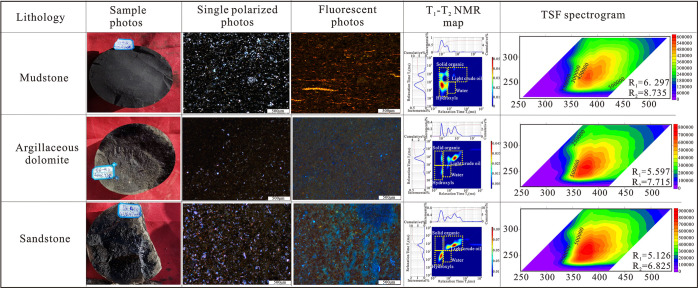
Dominant lithology analysis map.

The four light oil sweet spots all show a low organic matter content, but high light oil content, and have the characteristics of short-distance migration, forming a source-reservoir symbiosis. The corresponding lithology of the sweet spot section is argillaceous dolomite and sandstone, and the source rocks with a high TOC content are dominated by mudstone. The organic matter content from high to low is found in dolomitic mudstone, argillaceous dolomite, and sandstone, while the distribution of the light oil content is just the opposite, which indicates that sandstone and dolomite are good reservoirs, and the fluorescence color of sandstone and dolomite is blue, while that of mudstone is yellow, indicating that the oil quality in the reservoir is good and the mobility is strong. The oil in sandstone has the smallest *R*_1_ and *R*_2_, indicating that the crude oil has the lowest crude oil density. In addition, the TSF intensity is the highest, making it the best quality light oil reservoir, which is the same as the conclusions obtained by 2D NMR and fluorescent thin slices (Figure 13).

**Figure 14:**
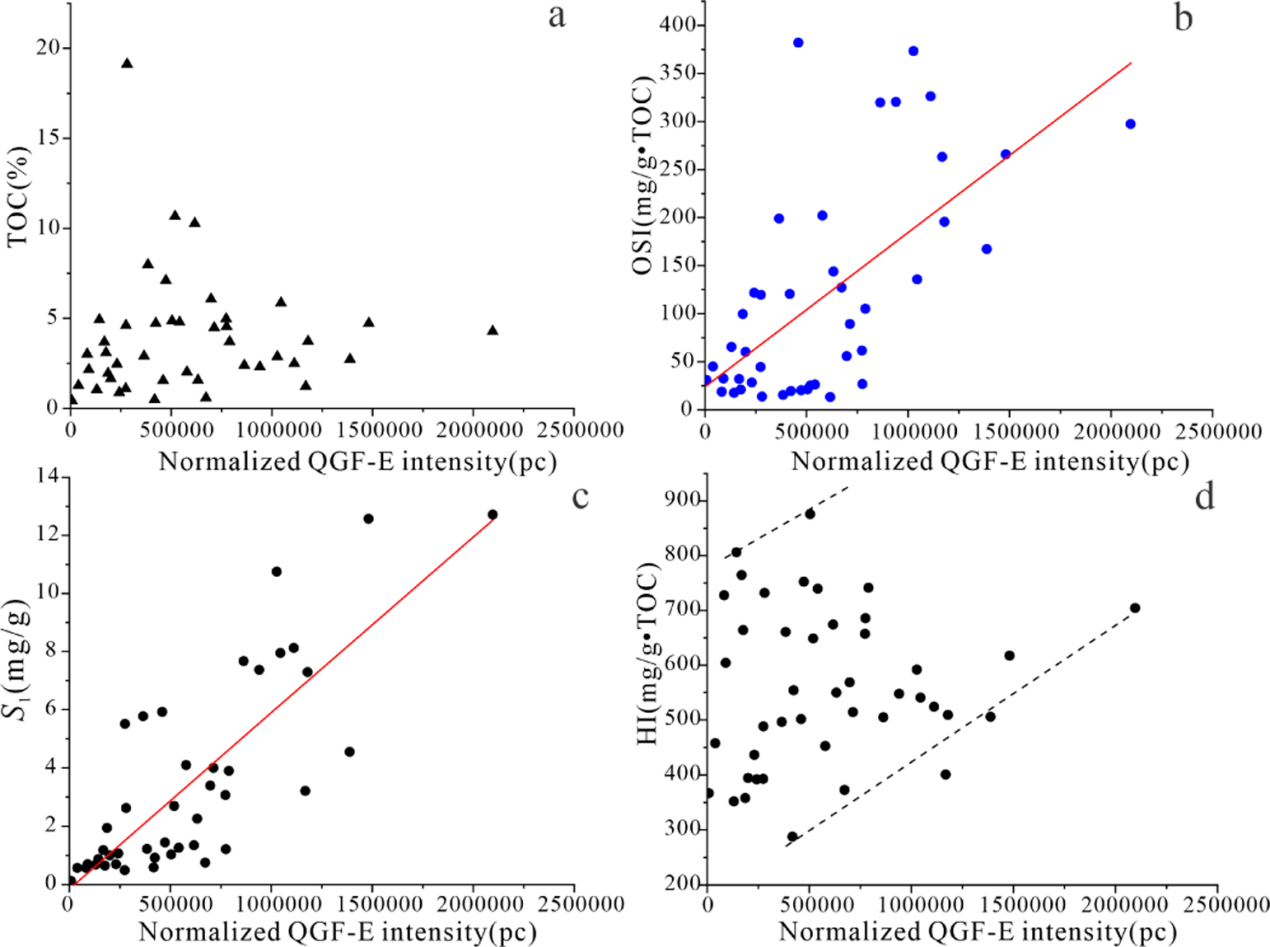
(a) Relationship between the normalized QGF-E intensity and TOC. (b) Relationship between the normalized QGF-E intensity and OSI. (c) Relationship between the normalized QGF-E intensity and *S*_1_. (d) Relationship between the normalized QGF-E intensity and HI.

There is no significant relationship between the QGF-E intensity and TOC content, but a weak positive correlation with HI and a significant positive correlation with OSI and *S*_1_, indicating that the contribution of micromigration oil to oil content is high (Figure 14). In order to further confirm the characteristics of oil short distance migration in the sweet spots of Lucaogou Fm in the study area, Li et al.^11^ took the sweet spots of Lucaogou Fm in well J174 as an example and carried out biomarker analysis of extracts from siltstone and its adjacent source rocks. The comparison of the results shows that they have a similar distribution.

Lian et al.^37^ pointed out that the source rocks of the Lucaogou Fm reached the peak of petroleum expulsion in Cretaceous/Paleogene, but the reservoir physical properties (porosity and permeability) were poor and oil viscosity was high, resulting in a large amount of oil retention and pore pressure increase. There is no light oil sweet spot in the lower part (burial depth more than 4366 m) due to the high content of heavy components, low content of free oil, poor physical properties, and insufficient residual fluid pressure.

#### 5.2.2 Light Oil Transportation Model

Although the average porosity of the Lucaogou Fm reservoir in the Jimusaer sag is 5.74%, which is higher than the corresponding porosity of the lower limit of the oil content (less than 5 or 4%, or even lower than 2%), it is an effective reservoir. However, the overall performance is still characterized by low porosity and low permeability (average permeability is 0.01 mD), which requires a certain initial pressure for oil migration. Previous studies have shown that the initial pressure of tight reservoirs in the study area is generally 0.16–0.37 MPa, and the maximum migration and accumulation power that buoyancy can provide is about 0.088 MPa, which is much lower than the power required for migration. However, the formation pressure of the Permian is generally higher than 30 MPa, and the pressure coefficient is generally greater than 1.2, which is in the overpressure zone.^38^ Therefore, the overpressure may be the main driving force for the short-distance migration of oil.^38^

The main causes of overpressure are diagenesis, under-compaction, and petroleum generation. With increasing diagenesis, smectite transforms into illite and releases a large amount of interlayer water and adsorbed water. However, the content of smectite and illite in the study area did not change significantly with the increase in burial depth (Table 1), and the smectite content is about 40%, indicating that diagenesis is not an important cause of overpressure. Lai^39^ verified that under-compaction is one of the important factors for the overpressure of the Lucaogou Fm in the study area by using the logging response characteristics of the formation (acoustic time difference of mudstone increases significantly, while lithology density and resistivity decrease). In addition, due to the good quality of source rocks in the study area, petroleum generation pressurization is also considered to be one of the important mechanisms for overpressure formation.^38^ The thermal simulation experiment of hydrocarbon generation shows that Lucaogou Fm has substantial oil generation potential (cumulative oil generation of 383.68 mg/g·TOC) and high efficiency of oil drainage (oil drainage rate is as high as 69.21%) in the low maturity stage (*R*_o_ < 0.8%), which results in the formation of large-scale low maturity oil.^40^ However, due to the characteristics of high viscosity of crude oil, short expulsion time, large thickness of source rock, and tight reservoir before hydrocarbon generation, the generated oil will be difficult to be expelled and can only migrate into reservoirs over a short distance. Continuous oil generation pressurization can be used as the driver of the short distance migration and accumulation. From oil generation simulation experiments on source rocks in limited space, the pressure of shale with a TOC value of 2.56% can be as high as 38 Mpa.^41^ Taking 4366 m as the boundary, the average TOC of the upper source rock is 3.56%, and that of the lower part is 4.74%. The generation pressurization intensity of the lower part is significantly higher than that of the upper part, and as a result, oil has a tendency to migrate from the bottom to the top.

**Figure 15:**
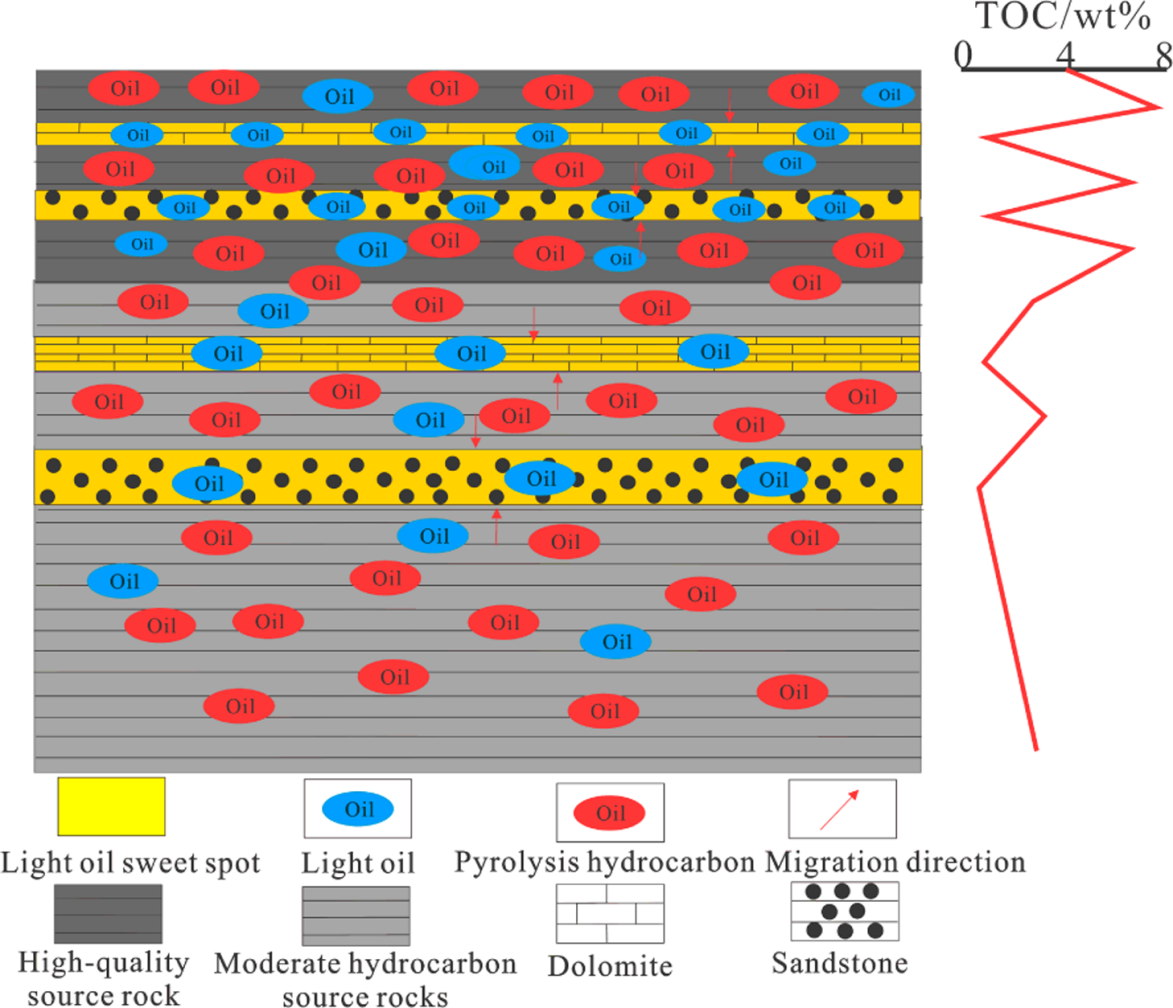
Shale oil accumulation model of well Ji-X.

In general, the source rock of the sweet spots in the Ji-X well has a good organic matter type and high petroleum generation potential. Under overpressure, light oils in the lower part have a tendency to migrate upward. There are four light oil sweet spots in the upper part. The oil generation and pressurization of shale lead to the accumulation of light oil in the adjacent reservoirs (sandstone and dolomite), forming a mode of “source-reservoir symbiosis” (Figure 15).^42^ In addition to overpressure as the driving force, good reservoir physical properties are also necessary for accumulation. The above four light oil sweet spots have high porosity and macro + mesopore contents (Figure 12). Therefore, if overpressure is matched with good reservoir physical properties, the sweet spot area can be formed.^43^ This kind of large-scale sustained petroleum generation pressurization (since the Late Jurassic) provides continuous power and material supply for oil accumulation, thus forming large-scale oil and gas resources.

## 6 Conclusions

The second part of the first Mbr of Lucaogou Fm in Jimusaer sag contains a set of fine-grained silty sand, mud, and carbonate sediments deposited in a saline lake environment. The lithology can be divided into seven types, with complex mineral compositions and strong substantial heterogeneity.

The overall oil saturation is relatively high, but the maturity of crude oil is low, and the overall oil quality is heavy, which is mainly controlled by the sedimentary environment and maturity of source rocks. Lithology and physical properties are the main controlling factors affecting the oil content. The high-quality source rock is dolomitic mudstone with a high organic content, and the high-quality light oil reservoir lithology is argillaceous dolomite and sandstone. Based on the fractal inflection point of the mercury injection curve, the pore space of the shale oil reservoir is divided into four categories, in which the higher the content of macropores and mesopores, the weaker the heterogeneity of the pore structure, and the better the oil content in the reservoir.

Taking the burial depth of 4366 m as the boundary, there are four light oil sweet spots in the upper part (burial depth less than 4366 m), and there are excellent source rocks with a high HI and a high organic matter content near each sweet spot. The pressurization caused by petroleum generation, and the formation of overpressured areas as a result, will drive the light oil to accumulate in high-quality reservoirs over short migration distances; there are source rocks with good petroleum generation potential in the lower part, but the content of heavy components is higher and the content of free oil is lower.
